# Performance Status Is a Predictive Factor of Improvement after Colonic Stenting in Patients with Malignant Stenosis due to Extraparenteral Malignant Tumors

**DOI:** 10.1155/2020/5931341

**Published:** 2020-06-27

**Authors:** Yasutoshi Shiratori, Naoki Ishii, Takashi Ikeya, Koichi Takagi, Kenji Nakamura, Katsuyuki Fukuda

**Affiliations:** ^1^Department of Gastroenterology, St. Luke's International Hospital, Tokyo, Japan; ^2^Department of Gastroenterology, Tokyo Shinagawa Hospital, Tokyo, Japan; ^3^Department of Gastroenterology, Tokyo Dental University Ichikawa General Hospital, Tokyo, Japan

## Abstract

Endoscopic stenting with self-expandable metallic stents is widely accepted for the treatment of malignant colorectal obstruction. Generally, stenting is performed as a bridge-to-surgery for primary colorectal cancer (CRC). Furthermore, palliative colonic stenting is performed for extraparenteral malignant tumors (EPMTs). However, predictive factors for improving clinical outcomes after colonic stenting for EPMTs remain unclear. This study is aimed at evaluating patients with EPMTs who underwent colonic stenting and investigating factors influencing clinical improvement after the stenting procedure. Twenty-one patients with colorectal obstruction by EPMTs were treated using self-expandable metallic stents over a period of 8 years. We divided the enrolled patients into the clinically improved and nonimproved groups after colonic stenting. Variables, including age; sex; type of primary cancer; serum albumin level; location of the obstruction; stent type, length, and diameter; prior chemotherapy treatment; ascites; Eastern Cooperative Oncology Group performance status (PS); technical and clinical success rates; stent-related adverse events; and mortality rates, were evaluated. Technical failure was not observed in all cases. Clinical success, defined as the passage of stool and improvement in the colorectal obstruction scoring system (CROSS) without adverse events, was observed in the 14 patients. Univariate analyses revealed no significant differences in factors other than PS before stenting (*P* = 0.04) between the improved and nonimproved groups. Colonic stenting for EPMTs was effective. PS may be an independent risk factor of clinical outcomes after stenting.

## 1. Introduction

Colorectal obstruction occurs in 8%-13% of patients with colorectal cancer (CRC) [1]. Before the development of endoscopic self-expandable metallic stents (SEMSs), emergency surgeries were performed to treat colorectal obstruction. Postoperative adverse events, associated with temporary or permanent colostomy, were common and negatively affected patients' quality of life. Generally, colonic stenting acts as a bridge-to-surgery (BTS) for primary CRC or as a palliative treatment of obstruction caused by extraparenteral malignant tumors (EPMTs), including metastases from primary tumors in the pancreas, breast, gynecologic organs, and stomach [2].

Colonic stenting is effective to avoid emergency surgery and improve abdominal symptoms. However, predictive factors for improving clinical outcomes after colonic stenting for EPMTs remain unclear. This retrospective cohort study is aimed at evaluating patients with colorectal obstruction by EPMTs who underwent colonic stenting and investigating the factors that affect clinical improvement after stenting.

## 2. Materials and Methods

### 2.1. Patients

**Figure 1:**
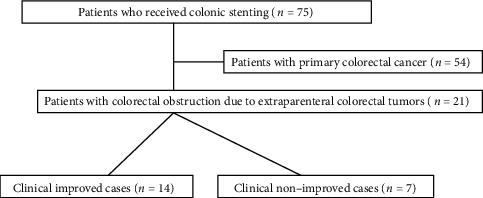
Study flow diagram depicting selection of cases. A total of seventy-five patients underwent colonic stenting for colorectal obstruction in 2 tertiary hospitals in Tokyo, Japan. Twenty-one patients with colorectal obstruction due to extraparenteral malignant tumors were included this study. We divided the patients into the clinically improved and nonimproved groups.

This was a retrospective analysis of a database used at St. Luke's International Hospital and Tokyo Shinagawa Hospital, Tokyo, Japan. Patients with a tumor growth < 4 cm in size from the anal verge, active bleeding, peritonitis, obstruction at multiple locations, and performance status (PS) 4 were excluded from the indication for colonic stenting. Out of the 75 patients who underwent colonic stenting, those with colorectal obstruction due to primary CRC were also excluded. A total of 21 patients with colorectal obstruction by EPMTs aged 56-86 (median, 71) years and treated with SEMSs between January 2011 and December 2018 at our hospitals were enrolled in the study. The poststenting follow-up period ranged from 46 to 2029 days, with a median of 247 days. We evaluated the colorectal obstruction scoring system (CROSS; Table 1) before and after stenting [3]. We collected data on age; sex; type of primary cancer; serum albumin level; location of obstruction (right, ascending and transverse colon; left, descending to the rectum); stent type (WallFlex or Niti-S), length, and diameter; prior chemotherapy treatment; ascites; Eastern Cooperative Oncology Group performance status (PS; grade 0, full active; 1, restricted in physically strenuous activity; 2, ambulatory and capable of self-care but unable to carry out any work activities; 3, capable of only limited self-care; 4, completely disabled; and 5, dead) of patients before stenting. We also evaluated the technical and clinical success rates, events of death within 30 days, and adverse events. We divided the enrolled patients into the clinically improved (14/21) and nonimproved (7/21) groups and examined their data statistically (Figure 1). The clinically improved and nonimproved groups were defined based on whether the clinical success of colonic stenting was obtained or not.

This study was approved by the institutional review board (18-R116, Nov.8.2018), and patient consent was waived owing to its retrospective design.

### 2.2. Stenting Procedure

**Figure 2:**
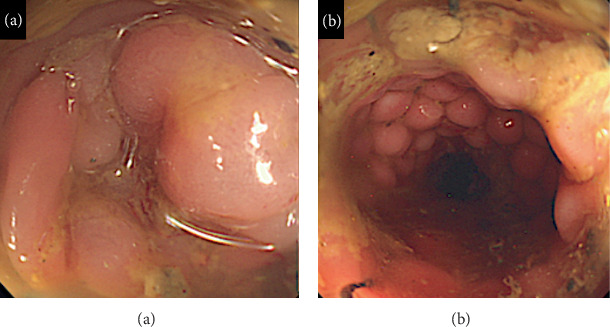
(a) Endoscopic findings before stenting, advanced tumor stenosis owing to primary descending colonic cancer. (b) Improved tumor obstruction and drainage obtained after stenting.

**Figure 3:**
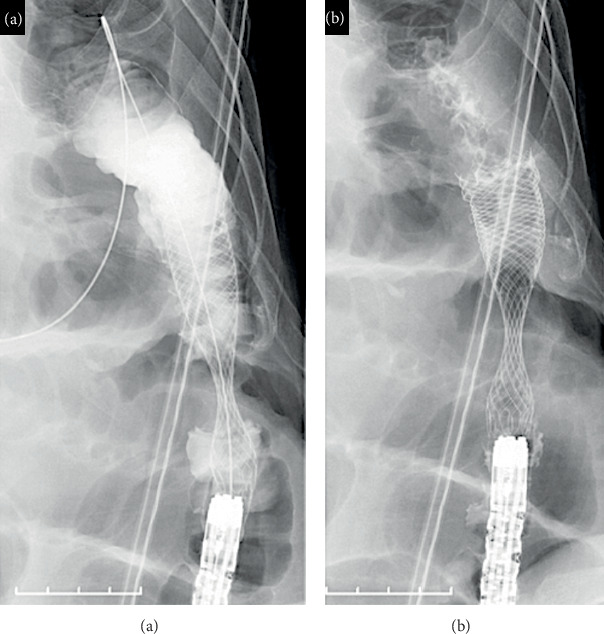
Colonography performed to confirm adequate placement of the stent. (a) We injected contrast agent through the endoscopy. (b) The contrast agent was naturally discharged.

All patients underwent computed tomography before stenting. The tumor margin was confirmed using fluoroscopy during the stenting procedure. A forward-viewing endoscope (GIF 260J or CF HQ290I; Olympus Optical Co., Ltd., Tokyo, Japan) was advanced to the tumor, and a marking clip was placed at the anal side of the tumor. A guidewire (0.035 inch, Boston Scientific) was passed inside the endoscopic retrograde cholangiopancreatography catheter and placed at the oral side of the tumor. The stent was placed along the guidewire. The type (WallFlex, Boston Scientific Co., Ltd., Natick, MA, USA; Niti-S, Taewoong Co., Ltd., Busan, Korea), diameter (18, 22, and 25 mm), and length (60, 80, 90, 100, and 120 mm) of the SEMS were selected by the endoscopist. Using colonography, we verified the position of the stent and whether the tumor was perfectly covered or not (Figures 2 and 3).

### 2.3. Definitions

Technical success of stenting was defined as the placement of stents across the entire length of colorectal malignant stenosis. Clinical success was defined as technically successful stent insertion, passage of stool, and improvement in CROSS without any procedure-related adverse events. CROSS was established by the Colonic Stent Safe Procedure Research Group, a research group of the Japan Gastroenterological Endoscopy Society, and its utility has been demonstrated [3]. Adverse events related to stenting were defined as adverse events that are mentioned in the American Society for Gastrointestinal Endoscopy lexicon [4] and occurred within 30 days after the procedure.

### 2.4. Statistical Analysis

Fisher's exact test was used for categorical variables. *P* values less than 0.05 were considered statistically significant. Statistical analyses were performed using Stata version 16 (StataCorp, USA).

## 3. Results

The patients' characteristics and procedures are summarized in Table 2. A total of 21 stents were placed. Technical success of stent placement was achieved in all patients. The rate of clinical improvement was 67% (14/21) of all patients. Clinical improvement was not observed in 33% (7/21) of patients. Adverse events related to stenting were seen in two patients: one with an early massive tumor bleeding and another with a perforation. No patients died within 30 days after stenting. The univariate analyses revealed no significant differences between factors, except for PS before stenting (*P* = 0.04) between the improved and nonimproved groups (Table 3).

## 4. Discussion

Colorectal obstruction is a life-threatening complication that requires immediate decompression. In patients with colorectal obstruction by EPMTs, palliative stenting was recommended [5]. Although several studies have reported the safety and effectiveness of SEMSs [6–9], colonic stenting can have a possible risk of potential complications. Hence, whether it is an effective procedure in patients with colorectal obstruction by EPMTs or not should be carefully evaluated.

We have demonstrated in a previous study that performance status was a predictive factor of dysphagia improvement after esophageal stenting in patients with malignant esophageal strictures and fistulas [10]. In this previous study, we indicated that a PS > 2 might be associated with cancer weakness and poor clinical improvement after esophageal stenting. The results of this previous study led to the hypothesis of the present study.

In this study, 21 patients with colorectal obstruction by EPMTs underwent SEMS placement. Technical success was achieved in all patients, and the clinical success was achieved at a rate of 67%. PS before stenting showed a significant difference between the improved and nonimproved groups in the univariate analysis. The patients in the PS = 0-2 group before stenting could have a high clinical success rate of 80% (12/15), and patients in the PS > 2 group could only improve by 33% (2/6). More patients in the PS = 0-2 group had higher clinical improvement, which was associated with longer survival after stent placement. We suggested that the group with PS > 2 was associated with cancer weakness and higher limitations of daily living that may reduce appetite due to colonic obstruction. In addition, we hypothesized that cancer pain, ascites, and distant metastases such as metastases from primary tumors in the liver and peritoneum, which are associated with decreased PS, were associated with nonclinical improvement. The evaluation of the causes of decreased PS is necessary. The complex influence of PS on the outcomes of this study needs to be further investigated.

A prior retrospective medical chart review reported that EPMTs showed a high risk of clinical failure, including perforation and death, in contrast to CRC [11]. The study also reported that radiotherapy was a significant predictor of endoscopic adverse events. In addition, a combination of multiple factors, such as radiotherapy and peritoneal metastasis, caused bowel immobilization that contributed to clinical failure and increased adverse events of colonic stenting in patients with EPMTs [11–13]. As there were no cases of radiotherapy prior to colonic stenting in our study, the confirmation of differences in this regard warrants further research.

In this study, 2 patients developed severe adverse events. One patient who underwent stenting for rectal obstruction due to urinary tract cancer invasion had massive hemorrhage 2 days after the procedure. We performed an emergency endoscopy, but active bleeding was not continuously seen during the procedure. We performed blood transfusion but did not need hemostatic interventions. The other complication was perforation: the patient had a sudden abdominal pain 7 days after the procedure. The patient developed a perforation of the rectal obstruction owing to peritoneal seeding of gastric cancer. The patient had no history of undergoing radiotherapy or chemotherapy. An emergency operation was performed after the diagnosis. The intraoperative findings showed a 7-8 mm perforation on the oral side of the stent. As the patient had no risk factors, such as prior radiotherapy, we suspected that the restriction of full-thickness wall extension by seeding was possibly attributable to the perforation. Furthermore, because the WallFlex stent was used in this patient, the axial force might have affected the colon. In terms of perforation, it is known that older age and location in the sigmoid colon are significantly associated with the occurrence of perforation and lower 30-day mortality rates [14].

Regarding the stent type, although a randomized prospective study has reported that ingrowth was more common with WallFlex stents than with other stents [15], we did not experience restenosis within 30 days in both cases of the WallFlex and Niti-S stents. The type of stents did not affect the clinical success and mortality rates in our study. Previous studies have reported that it was difficult to obtain clinical success if stenosis was long (>4 cm) [16]. However, we found that stenosis length was not a significant predictor.

This study had several limitations. First, this was a retrospective study with all of the inherent limitations of a retrospective study. Second, the stent type, diameter, and length varied in patients. Third, this study included a small sample size. Fourth, this study did not include patients who underwent radiation therapy or carcinomatosis which might have affected the clinical improvement of stenting. However, the results presented are encouraging enough to warrant further prospective trials involving a larger number of patients with a longer duration of follow-up.

## 5. Conclusions

Our findings suggest that PS is an independent factor for clinical improvement in patients with colonic stenosis due to EPMTs after stenting. Colonic stent placement is a procedure with complications, and gastroenterologists might need to think about stenting adaptation with reference to PS.

## Figures and Tables

**Table 1:** The colorectal obstruction scoring system.

Level of oral intake	Score
Requiring continuous decompressive procedure	0
No oral intake	1
Liquid or enteral nutrient	2
Soft solids, low residue, and full diet with symptoms of stricture	3
Soft solids, low residue, and full diet without symptoms of stricture	4

**Table 2:** Patient and procedural characteristics.

Age	Sex	Primary cancer	Albumin (g/dL)	Location	Stent	Stenosis length (cm)	Stent length (mm)	Stent diameter (mm)	Chemotherapy	PS	CROSS (pre)	CROSS (post)	Adverse events
57	F	Breast	3.5	R	Wall	5	90	22	+	1	2	4	None
72	F	Breast	4.1	D	Niti-S	6	100	18	-	1	1	3	None
74	F	Breast	2.8	R	Niti-S	3	60	25	+	1	2	4	None
77	M	Stomach	2.2	T	Niti-S	5	100	18	-	2	2	3	None
86	F	Stomach	2.2	R	Wall	6	90	18	-	3	1	1	None
76	M	Stomach	3.1	R	Wall	6	120	18	-	1	1	3	None
59	F	Stomach	2.9	R	Wall	6	90	18	-	3	1	1	Perforation
81	F	Stomach	2.1	S	Niti-S	5	100	22	-	1	2	4	None
69	M	Stomach	3.4	S	Niti-S	6	100	18	-	3	1	3	None
84	M	Stomach	2.7	S	Niti-S	4	80	22	-	3	2	4	None
78	M	Stomach	2.6	R	Niti-S	6	100	22	-	2	1	3	None
56	F	Ovary	2.4	R	Niti-S	4	80	22	-	3	1	3	None
56	F	Ovary	2.8	R	Niti-S	4	80	18	-	3	1	1	None
52	F	Ovary	2.8	R	Niti-S	6	100	22	-	1	1	3	None
68	F	Ovary	3.1	S	Niti-S	6	100	18	-	1	1	3	None
74	F	Ovary	2.3	D	Niti-S	6	100	18	+	0	0	0	None
65	M	Pancreas	3.7	S	Niti-S	6	100	22	+	1	0	2	None
69	M	Pancreas	2.9	R	Wall	5	90	22	+	1	0	0	None
81	M	Urinary	3.4	S	Wall	6	120	22	+	1	1	1	None
73	M	Urinary	2.8	R	Wall	4	90	22	-	1	1	3	Hemorrhage
76	M	Urinary	4.2	D	Niti-S	4	80	22	-	1	1	4	None

PS: performance status; CROSS: colorectal obstruction scoring system; M: male; F: female; T: transverse colon; D: descending colon; S: sigmoid colon; R: rectum; Wall: WallFlex colonic stent (Boston Scientific Co., Ltd., MA, USA); Niti-S: Niti-S colonic stent (Taewoong Co., Ltd., Busan, Korea).

**Table 3:** Univariate analysis of factors for clinical improvement.

	Improvement (*n* = 14)	Nonimprovement (*n* = 7)	*P* value
Age (≧70, <70)	8, 6	4, 3	0.99
Sex (M, F)	7, 7	3, 4	0.75
Albumin (≧2.5, <2.5)	11, 3	5, 2	0.46
Location (right, left)	1, 13	0, 7	0.26
Stent (Wall/Niti-S)	4, 10	3, 4	0.53
Length of stenosis (>4 cm, ≦4 cm)	9, 5	3, 4	0.42
Stent diameter (≧22 mm, <22 mm)	8, 6	4, 3	0.43
Chemotherapy (+, -)	3, 11	3, 4	0.42
Ascites (+, -)	3, 11	4, 3	0.35
Pre-PS (≦2, >2)	12, 2	3, 4	0.04
Adverse events (+, -)	0, 12	2, 5	0.03

PS: performance status; M: male; F: female; Wall: WallFlex colonic stent (Boston Scientific Co., Ltd., MA, USA); Niti-S: Niti-S colonic stent (Taewoong Co., Ltd., Busan, Korea).

## Data Availability

The endoscopic procedure data used to support the findings of this study are included within the article.
